# A Transcriptional Signature for Active TB: Have We Found the Needle in the Haystack?

**DOI:** 10.1371/journal.pmed.1001539

**Published:** 2013-10-22

**Authors:** Adithya Cattamanchi, Nicholas D. Walter, John Z. Metcalfe, J. Lucian Davis

**Affiliations:** 1 Division of Pulmonary and Critical Care Medicine, San Francisco General Hospital, University of California San Francisco, San Francisco, California, United States of America; 2 Curry International Tuberculosis Center, San Francisco General Hospital, University of California San Francisco, San Francisco, California, United States of America; 3 Division of Pulmonary Sciences and Critical Care Medicine, University of Colorado Denver, Aurora, Colorado, United States of America

## Abstract

Adithya Cattamanchi and colleagues reflect on recent research by Michael Levin and coworkers into the use of whole blood mRNA expression signatures to detect tuberculosis. The authors highlight challenges faced in getting this promising technology into clinics in low-resource settings.

*Please see later in the article for the Editors' Summary*

**Linked Research Article**

This Perspective discusses the following new study published in *PLOS Medicine*:

Kaforou M, Wright VJ, Oni T, French N, Anderson ST, et al. (2013) Detection of Tuberculosis in HIV-Infected and -Uninfected African Adults Using Whole Blood RNA Expression Signatures: A Case-Control Study. PLoS Med 10(10): e1001538. doi:10.1371/journal.pmed.1001538.

Using a microarray-based approach, Michael Levin and colleagues develop a disease risk score to distinguish active from latent tuberculosis, as well as tuberculosis from other diseases, using whole blood samples.

Analysis of whole-genome RNA expression in human clinical samples is a relatively novel approach to biomarker development. The pattern of RNA expression (i.e., transcriptional signature) can provide a “biological snapshot” of the immune response to physiological stressors, and specific disease states may produce distinct transcriptional signatures. In this week's issue of *PLOS Medicine*, Michael Levin and colleagues report that a blood RNA transcriptional signature can be used to diagnose active tuberculosis (TB) in high HIV/TB prevalence settings. Using blood samples from patients referred for TB evaluation at three sites in Cape Town, South Africa (cases from an outpatient TB clinic; controls from two hospitals) and one district hospital in Northern Malawi, the authors identified a minimal set of 44 transcripts that distinguished patients with TB from patients confirmed to have an alternative diagnosis. They then converted the complex expression data into a simple-to-calculate disease risk score, which was highly sensitive (93%, 95% CI [83–100]) and specific (88%, 95% CI [74–97]) for active TB.

## Have We Found the Needle in the Haystack?

This landmark study advances the field in several critical ways. For the first time, a blood transcriptional signature for TB was defined by comparison with patients who have conditions that mimic TB in a high burden setting instead of with healthy controls or patients with sarcoidosis or auto-immune diseases [1]–[4]. The inclusion of controls for whom TB was in the list of differential diagnoses but ultimately excluded increases confidence that the blood transcriptional signature identified may be clinically relevant. Second, the authors developed the disease risk score, which provides a single measure of the degree to which an individual's RNA expression is consistent with TB. The disease risk score can be calculated by simply subtracting the summed normalized intensities of down-regulated transcripts from those of up-regulated transcripts. By eliminating the need to use complicated bioinformatics to make predictions from the RNA expression data, the disease risk score could simplify the application of transcriptional signatures in clinical settings. Finally, the authors demonstrate convincingly the high diagnostic accuracy of their blood transcriptional signature. The results were impressive in their test set (20% of enrolled patients), including in HIV-infected and smear-negative sub-populations, and in an entirely independent validation dataset published years earlier [1].

Although the results are promising, key questions remain. First, can the results be reproduced in a truly representative population? State-of-the-art technology and bioinformatics are critical tools for identifying prospective targets, but the rigorous application of fundamental epidemiological principles will be indispensable to advancing these technologies into the clinical arena, where it will be necessary to show their utility in truly representative populations. Levin and colleagues describe an “intention-to-test” recruitment strategy but nonetheless enrolled a highly selected patient population. TB patients generally had advanced disease (over 90% of HIV-uninfected patients [96/106] and over 75% of HIV-infected patients [83/109] were smear-positive) and 28% (207 of 751) of patients were excluded because TB status was uncertain. The control group was recruited entirely from inpatient wards and most patients had non-respiratory diseases. These factors resulted in a spectrum bias towards the extremes of disease manifestations, which is known to inflate estimates of diagnostic accuracy [5]. Second, can a robust threshold be developed for the disease risk score that works in different settings? An inherent limitation of the microarray technology used in this study is that it provides only relative quantification of RNA expression so that intensity values are relevant only within, and not across, experiments [6]. Ultimately, to be clinically useful, a threshold will need to be defined *a priori* rather than on the basis of experimental data and the selected threshold will have to be consistent across geographic settings. Finally, can a platform be developed to enable measurement of the transcriptional signature in low-income countries? A number of novel technologies for quantitative multi-channel measurement of nucleic acid targets are in development. However, the cost and difficulty of assaying a 44-transcript signature seem prohibitive far into the future. Absent a transformative technology, it is difficult to envision transcriptional profiling having a meaningful impact in parts of the world where novel TB diagnostics are most needed [7].

## Triage Testing: A Target for Future Research

As Levin and colleagues suggest, their 44-transcript DRS may be more useful as a triage (i.e., rule-out) test [8] because negative predictive value is high (98%, 95% CI [96–100]), while positive predictive value is sub-optimal (66%, 95% CI [46–87]) when TB prevalence is 20%, as is common in routine settings. The concept of a triage test deserves further attention in the TB diagnostics literature. An ideal triage test rules out disease when negative and triggers further testing when positive (e.g., a mammogram for breast cancer screening). Thus, a triage test requires near-perfect sensitivity (particularly when the consequence of missing disease is high) but only moderate specificity. If rapid and inexpensive, such a test could be used to determine which patients presenting with TB symptoms require confirmatory testing (e.g., automated nucleic acid amplification testing or culture) and for TB screening as is recommended in high-risk populations including people living with HIV and household contacts of active TB cases [9],[10].

The 44-transcript signature identified by the authors shows promise as a triage test and might be further optimized for this purpose during its further development. Indeed, it is likely that any set of host-derived biomarkers, especially if based on generic rather than antigen-stimulated immune responses, will have more difficulty achieving high specificity than high sensitivity. In this regard, the commonly used approach of selecting a diagnostic threshold that maximizes the number of correctly classified outcomes is misguided [11]. This cannot lead to a clinically useful test if neither sensitivity nor specificity is high enough to provide meaningful rule-out or rule-in value. Future work to establish a threshold for the 44-transcript signature as a triage test should focus on maximizing sensitivity, even at the cost of decreased specificity. Furthermore, focusing on developing a triage test at the discovery stage may lead to selection of a different set of transcripts that retains 100% sensitivity while providing even better specificity.

## Summary

Levin and colleagues have provided compelling proof of the concept that a blood transcriptional signature can distinguish between TB and clinical mimics in high-incidence settings. The field can now move on to asking more practical questions to determine the feasibility and optimal use for an RNA expression-based biomarker for TB in clinical settings. Finding the right signature—the proverbial “needle in the haystack”—may require additional discovery work involving smaller sets of RNA transcripts and will certainly require validation of candidate signatures in diverse settings. Further discovery and validation studies should adhere to fundamental principles of high quality diagnostic evaluations [12], including enrollment of consecutive patients presenting for TB evaluation in representative health facilities. Even if validated, significant technical hurdles remain to translate these important findings to rapid, inexpensive, and simple assays that can impact patient outcomes in countries where TB is most prevalent. The search continues.
